# Effects of gold and copper mining on the structure and diversity of the surrounding plant communities in Northeast Tiger and Leopard National Park

**DOI:** 10.3389/fpls.2024.1419345

**Published:** 2024-06-11

**Authors:** Xue Wang, Yue Li, Xueyuan Bai, Lianxi Sheng, Houling Zhang, Faping Chen, Yujun Xiao, Wenze Liu, Yuquan Zhai

**Affiliations:** ^1^ State Environmental Protection Key Laboratory of Wetland Ecology and Vegetation Restoration, School of Environment, Northeast Normal University, Changchun, China; ^2^ Key Laboratory for Vegetation Ecology, Ministry of Education, Northeast Normal University, Changchun, China; ^3^ Key Laboratory of Environmental Materials and Pollution Control, The Education Department of Jilin Province, College of Engineering, Jilin Normal University, Siping, China; ^4^ Hunchun Zijin Mining Limited Company, Environmental Protection Department, Hunchun, China

**Keywords:** Northeast Tiger and Leopard National Park, mining, plant community structure, species diversity, species distribution

## Abstract

**Introduction:**

Northeast China Tiger and Leopard National Park is home to the largest and only breeding family of wild tigers and leopards in China. The mining of open-pit gold and copper mines in the core zone might affect the surrounding forest ecosystem and the survival activities of wild tigers and leopards.

**Methods:**

In order to understand the impacts of gold and copper mining on the structure and diversities of the surrounding plant communities, the vegetation of the forest layer, shrub layer and herb layer of the forest community in the original forest area, mining area, tailings area and restoration area of the Northeast China Tiger and Leopard National Park were investigated, and the influence of plant community structure on species diversity was also evaluated.

**Results:**

This study concluded that there are 25 species belonging to 11 families, 16 genera of trees, 43 species belonging to 22 families, 35 genera of shrubs, and 57 species belonging to 23 families, 46 genera of herb in the sampling sites. There were no significant differences in the community structure characteristics and species diversities of the tree layer and the shrub layer in different operational areas. However, in herb layer, the heights, the coverage and the species diversity index were higher in the restoration area. Additionally, the community structure was one of the major factors that influence the diversity indices, which might be an important way for mining to impact plant diversity.

**Discussion:**

Therefore, mining had some impacts on the structure and diversity of the surrounding plant communities, but the impacts did not reach a significant level. These results could provide scientific support for the management of the forest ecosystems around the mining area of Northeast Tiger and Leopard Park.

## 1 Introduction

The community structure and species diversity play an important role in evaluating the function of forest ecosystems (Wei et al., 2024), and a stable community structure is the foundation of forest ecosystem services (Li et al., 2023), while plant diversity can quantitatively characterize plant communities and ecosystems (Hooper et al., 2005). Changes in diversities can reflect the heterogeneity of community composition, structure, and function, which are the basis and indicators for maintaining ecosystem service functions (Nguyen et al., 2012; Pan et al., 2018).

Recently, people have continuously transformed natural ecosystems into agricultural and industrial production systems that can be utilized by humans, which has caused great threats to species diversities and even led to the degradation of ecosystems (Nyström et al., 2019). Among these human interference, mining could change plant community structure by damaging plants and disturbing plant growth. Open pit mines might change the direction of the succession of plant communities, and the community structure might tend to be simpler, and the vegetation cover and density declined in the mining area compared with the non-mining area (Kayet et al., 2020; Lloyd et al., 2023). Additionally, mining activities will cause the groundwater level of the submersible aquifer to decline with the increase of the extraction space, which ultimately leads to a decrease in the amount of groundwater resources (Qiao et al., 2011; Shi et al., 2017). The established and dominant species usually cannot adapt to this environmental change, and are gradually replaced by new pioneering species, then plant community succession occurs (Sheil, 1999; Catford et al., 2012). Moreover, the distance from the mining area is also a key factor affecting the characteristics of plant community structure, as the distance from the mining area decreases, the ecosystem degradation phenomenon is visible, and the production function is also gradually reduced (Zeng et al., 2024), the further away from the mining area, the closer the plant community is to the natural ecosystem. However, Zou et al. (2019) came to a different result, they found open-pit mining did not have a significant impact on the surrounding communities, and that mining activities were not the key reason of degradation of the surrounding grassland ecosystems (Zou et al., 2019). In summary, more cases are needed to verify whether mining will have a significant impact on the community structure of ecosystems.

Open-pit mining seriously interferes with the plant diversity in ecosystems (Yang et al., 2023), which has also caused great concern. Compared with non-mining areas, the richness and diversity indices of plant communities in mining areas significantly decrease (Wang et al., 2014; Yang et al., 2023), and subsequently affects the stability of ecosystems. Firstly, open-pit mining might cause the decline of the groundwater level and further reducing the soil moisture (Luan et al., 2020), and then resulting in a decrease in the number of species in the community (Deng et al., 2016). Secondly, mining might also lead to the loss of the soil nutrients (Swab et al., 2020) that are usually correlated with plant diversity (Han et al., 2019). In addition, plant diversity also correlates with the degree of the disturbance of open-pit mining, and high species richness usually occurs in low-disturbed ecosystems (Seidl et al., 2022). Open pit mining not only decreases the species diversity, also leads to a decrease in the dominant species and an increase in the plant community evenness index. Moreover, the species evenness increased with the decreasing distance from the mining area. However, the effects of open pit mining on plant community structure and diversity are mostly focused on grassland ecosystems. There is a relative lack of research on forest ecosystems. Therefore, it is more important to strengthen the research on the impacts of mining on the species diversity of forest ecosystems for biodiversity conservation.

Additionally, mining might affect plant diversity through influencing species composition. The interrelationship between forest community structure and diversity is the basis of forest management, and it has been found that forest community structure (including height, diameter at breast height (DBH), crown width, cover, etc.) also affect the growth, diversity of plant in forest ecosystems (Li et al., 2023). Király and Ódor (2010) conducted a study in mountain forests of western Hungary and found tree diameter at breast height can significantly influence species composition and diversity in the understory (Király and Ódor, 2010). Studies have shown that forest density can influence light, penetration, temperature and humidity in forest (Chen et al., 2020; Cui et al., 2022), resulting in changes in the survival of understory plants. Therefore, the diversity of some of the understory plants (e.g. vascular plants) would decrease with the increasing canopy density due to the shade (Schoonmaker and McKee, 1988; Jiménez et al., 2015). As a result, clarifying the coupling relationship between forest ecosystem community structure and species diversity can provide theoretical support for forest ecosystem function enhancement and management. However, researches mainly focus on undisturbed natural forests, and research on the coupling relationship between plant community structure and diversity disturbed by open-pit mining still needs further explored.

Northeast Tiger and Leopard National Park is the home to the largest and only breeding population of wild northeastern tigers and leopards in China. Northeast tigers and leopards are the top of the food chain of the forest ecosystem, which are signs of ecological health, and their survival is closely related to their habitat. Thus the protection of the plant community structure of the forest ecosystem and the maintenance of the stability of the ecological service function are of great importance for the survival of the wild tigers and leopards. The mining of open-pit gold and copper mines in the core protected area of the Northeast Tiger and Leopard National Park might have a certain impact on the surrounding forest ecosystems and the survival of wild tigers and leopards. Moreover, tailings, slag and other wastes discharged during the mining process have seriously disturbed the surrounding ecosystems, causing drastic changes in the soil environment and a serious decline in soil fertility. Mining could also threaten the growth and development of plants and trigger the change of community structure and functional degradation of plant communities. To our knowledge, previous studies of the park have focused on the protection of wild animals and the development of eco-tourism, but the ecological impacts of open-pit mining on the plant community structure and species diversity of the trees, shrubs and herbs in the habitat of wild tigers and leopards is still lack of research. Therefore, investigating and analyzing the plant community structure and diversity of forest ecosystems are crucial for the survival and conservation of wildlife such as the northeastern tiger and leopard. In this study, the open-pit gold and copper mine in the core area of the Northeast Tiger and Leopard National Park was used as the research object to investigate the impacts of mining on the structure and diversity of the surrounding forest communities, and provide scientific support for the protection and management of the Northeast tiger and leopard habitats in the future.

## 2 Materials and methods

### 2.1 Study sites

**Figure 1:**
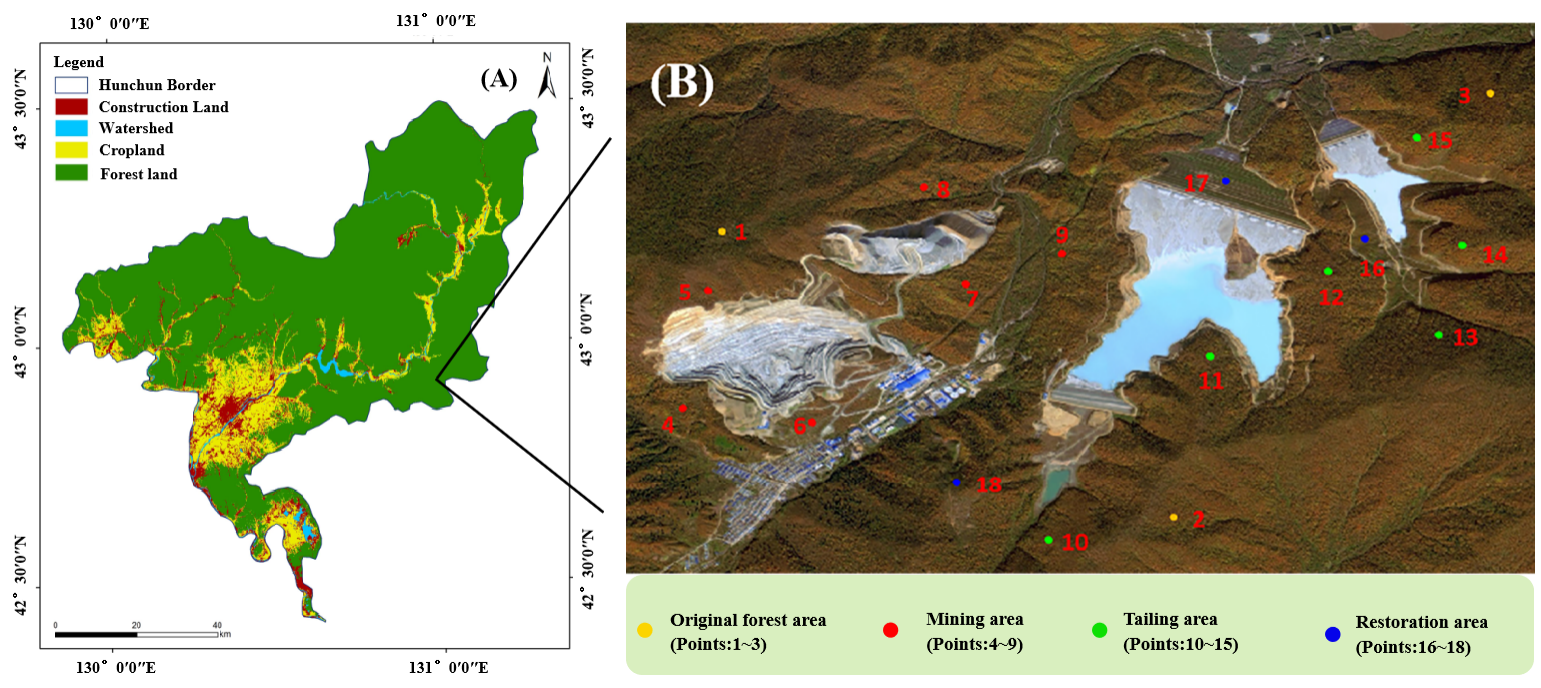
**(A)** Hunchun National Tiger and Leopard Park, **(B)** Sampling sits of Shuguang gold and copper mine (figure A was modified based on Hunchun Resource Survey, Jilin, China, http://www.jl1.cn/xuanchuan3_view.aspx?id=135).

Shuguang open-pit gold and copper mine is located in the core area of Northeast Tiger and Leopard National Park, the eastern part of Hunchun (42°38′45″~44°18′36″ N, 129°05′01″~131°18′52″ E) (**Figure 1A**). The mine was established in January 2003, with a total annual output of 8.25 million tons of raw ore and waste rock that can be comprehensively utilized. The mine is a semi-depressed open-pit mine on a semi-hillslope, divided into South Mountain Pit and North Mountain Pit. The main source of ore production is the open-pit quarry on the North Mountain Pit, and the mining in the South Mountain Pit is only used as a by-production field due to the resource conditions. The Northeast Tiger and Leopard National Park became pilot in 2017 and was formally established in 2021. The region is characterized by low meso-mountainous geomorphic area, low hilly geomorphic area and river valley and basin geomorphic area. The climate belongs to the offshore Monsoon Climate of Medium Latitudes, which is characterized by four distinct seasons, cold winters and hot summers, and synchronized rain and heat. The temperature varies significantly under the influence of the oceanic climate. The typical zonal soil is dark brown soil, with a small amount of swampy soil and alluvial soil. The soil-forming parent material is acidic rock weathering material, and the soil is slightly acidic.

### 2.2 Survey of species diversities

Four sampling sites were set up in the experiment, including original forest area, mining area, tailing area and restoration area, totally 18 sampling points (**Figure 1B**). Among the samping sites, point 16 to 18 were sampling sites that have been restored for ten years, 5 years and 20 years, respectively. Plant diversity investigation around the mine area was conducted in July 2022 during the peak plant growth season. The sample squares of trees, shrubs and herbs were 20 m × 30 m, 10 m × 10 m, and 1 m × 1 m, respectively. In order to investigate the plant community composition, the name, number, height, crown width (cover) were also recorded.

### 2.3 Measurements of the parameters of plant diversity and plant community structure

The plant community structure, plant diversity and variability of plant communities in the Northeast Tiger and Leopard National Park were calculated respectively, and the details of the indexes and the calculation methods used are shown in **Table 1**.

**Table 1:** Methods of plant community structure, plant diversity, and variability.

Index	Methods
Indexes of plant community structure	
Density	Number of plants per unit area.
Relative Density	Percentage of the number of individuals of a species in a sample plot over the number of individuals of all species.
Coverage	Area covered by the base of the plant per unit area. Trees are calculated as the area broken at breast height (at 1.3 m).
Relative Coverage	Percentage of the sum of the sub-coverage of a species in a community.
Height	The length of a plant body. The measurement is taken at its natural height.
Relative Height	Percentage of the height of a species over the height sum of all trees in the sampling site.
Important Value	(Relative density + relative dominance + Relative height)/3
Diameter at breast height	The diameter of the tree trunk at the chest height above the ground surface (usually expressed in cm), measured as the average of the maximum and minimum values when the cross-section is deformed.
Biodiversity indexes	
Species Richness	*R* = *S* Where *R* represents species richness index, *S* represents the number of species in each sample plot.
Shannon- Weiner index	$H=-\sum_{i=1}^{S}P_{i}\text{In}\;\left(P_{i}\right)$ Where *H* represents the species diversity index, *S* represents the number of species in the plant community, and *Pi* is the proportion of the *i*_th_ species in the total number of species in each sample plot.
Simpson Index	$D=1-\sum_{i=1}^{s}\;P_{i}^{2}$ Where *S* represents the number of species in the plant community, and *Pi* is the proportion of the *i*_th_ species in the total number of species in each sample plot.
Evenness Index	$J_{sw}=\frac{-\sum_{i=1}^{s}P_{i}\;\text{In}\;\left(P_{i}\right)}{\text{In}S}$ Where S represents the number of species in the plant community, and *Pi* is the proportion of the *i*_th_ species in the total number of species in each sample plot.
Alatalo Index	$E_{a}=\frac{\frac{1}{\sum_{i=1}^{S}P_{i}^{2}}-1}{\text{exp}\;\left(-\sum_{i=1}^{S}P_{i}\;l\text{n}P_{i}\right)-1}$ Where *S* represents the number of species in the plant community, and *Pi* is the proportion of the *i*_th_ species in the total number of species in each sample plot.
**Variability indexes:** variability indexes represent the range of variation of community structure indexes, and are often expressed in terms of standard deviation and coefficient of variation (CV).	
Tree layer	The range of variation of community structure indexes of the heights, diameters at breast height (DBH) and crown spread in the tree layer.
Shrub layer	The range of variation of community structure indexes of tree height, basal diameter and crown spread in the shrub layer.
Herb layer	Height

### 2.4 Statistical analysis

All data analyses were performed using SPSS 22.0 (IBM Corporation, Chicago, IL, USA) software. One-way ANOVA was used to analyze the structure and diversity of plant communities in different operational areas, and correlation was used to analyze the relationship between community structure indicators and plant diversity indexes.

## 3 Results

### 3.1 Distribution characteristics of dominant species in different operational areas

There are 11 families, 16 genera and 25 species of trees in the survey sample site, the details of the dominant species are showed in **Table 2**.

**Table 2:** Dominant species in different operational areas.

	Original forest	Mining area	Tailings area	Restoration areas
**Tree**	*Quercus mongolica* Fisch. ex Ledeb.	*Tilia amurensis* Rupr., Acer *pseudo-sieboldianum* (Pax.) Kom. and *Abies holophylla Maxim*	*Abies holophylla Maxim* and *Quercus mongolica* Fisch.	No trees were found
**Shrub**	*Rhododendron mucronulatum* Turcz., *Acer pictum* Thunb. and *Corylus mandshurica* Maxim. & Rupr.	*Syringa reticulata* subsp. amurensis (Rupr.) P. S. Green & M. C. Chang, *Euonymus verrucosus* Scop. and *Corylus mandshurica*	*Acer pseudo-sieboldianum* and *Schisandra chinensis* (Turcz.) Baill.	*Salix schwerinii* E. L. Wolf, *Rubus crataegifolius* and *Populus koreana* Rehder
**Herb**	*Maianthemum bifolium*(L.)F.W.Schmidt, *Scutellaria pekinensis* Maxim and *Carex meyeriana* Kunth	*Carex* and *Carex siderosticta* Hance	*Carex siderosticta* and *Carex siderosticta* Hance	*Poa annua* L., *Brachybotrys paridiformis* Maxim. ex Oliv., and *Rubia chinensis* Regel et Maack

### 3.2 Community structure in different operational areas

The details of the community structure characteristics of the sample plots are shown in **Table 3**. The maximum values of the weighted average indices of tree height, DBH and crown width were all found in the original forest, and the minimum values were all found around the mining area. There are no significant differences in the community structure characteristics of the tree layer and the shrub layer in different operational areas. In addition, in the shrub layer, the crown diameter in the restoration area is higher than that in the original forest and other operational areas. Similarly, the height and coverage in the herb layer are the largest in the restoration area compared with other operational areas, and the minimum values occurred at original forest (*P<* 0.05) (**Table 3**).

**Table 3:** Characterization of plant community structure in tree, shrub and herb layers in different operational areas.

		Density (pcs/ha)	Height (m)	DBH (m)	Crown diameter (m)
**Tree**	Original forest	394 ± 77	9.67 ± 0.922	18.52 ± 4.861	5.69 ± 1.331
	Mining area	469 ± 151	8.48 ± 1.126	10.96 ± 4.906	4.68 ± 0.433
	Tailings area	312 ± 9	9.32 ± 0.092	19.60 ± 1.166	5.64 ± 0.058
		Density (pcs/ha)	Height (m)	Base diameter(cm)	Crown diameter (m)
**Shrub**	Original forest	2971 ± 572	0.68 ± 0.233	0.87 ± 0.337	0.42 ± 0.113^ab^
	Mining area	4765 ± 1292	0.78 ± 0.544	0.94 ± 0.827	0.51 ± 0.323^ab^
	Tailings area	4792 ± 1789	0.40 ± 0.048	0.51 ± 0.065	0.31 ± 0.058^b^
	Restoration areas	3151 ± 1029	3.36 ± 2.826	1.87 ± 1.063	1.19 ± 0.630^a^
		Density (pcs/ha)		Height (m)	Coverage (%)
**Herb**	Original forest	76905 ± 7841		0.09 ± 0.042^b^	19.90 ± 17.75^b^
	Mining area	81667 ± 45200		0.22 ± 0.033^ab^	37.87 ± 16.22^b^
	Tailings area	80139 ± 19437		0.14 ± 0.047^b^	49.43 ± 13.05^ab^
	Restoration areas	108667 ± 54347		0.37 ± 0.176^a^	99.73 ± 41.27^a^

Lowercase letters represent significant levels of difference (P< 0.05).

### 3.3 Species diversity in different operational areas

**Figure 2:**
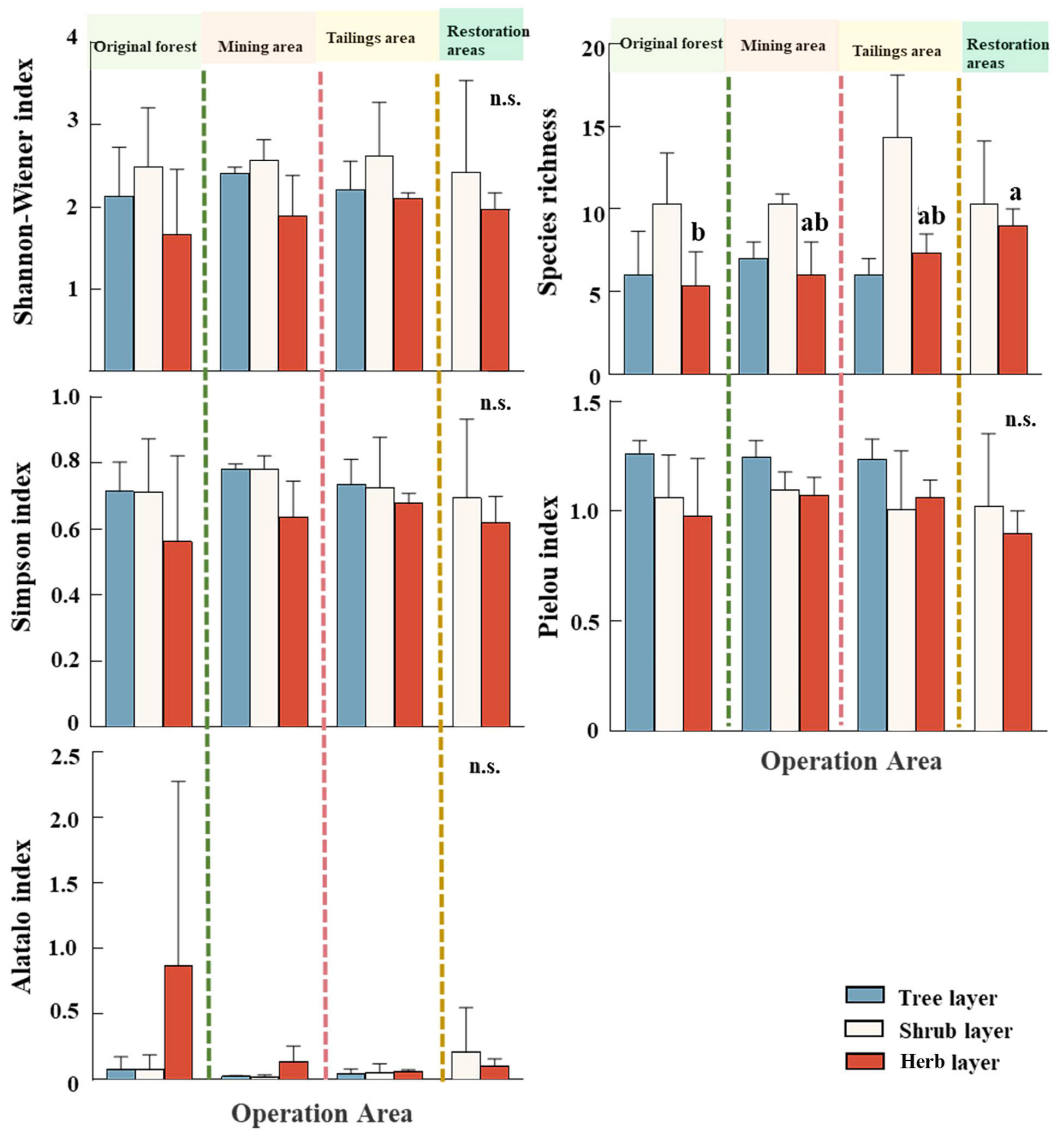
Diversity indices for each operational area in the tree, shrub, and herb layers, lower case letters (i.e. a, ab, b) indicate significant difference test by ANOVA, 'n.s.' means 'not significance'.

Species diversity indices in the study area showed some variabilities. The diversity indices (Shannon Wiener, richness and Simpson indices) were higher in mining and tailing area compared with that in the original forest and restoration area in the tree layer and shrub layer, while the differences in evenness indices were subtle. And the difference in species diversity index between different operating areas did not reach a significant level (*P* > 0.05). Additionally, species richness in the herb layer presented significant differences in four investigation areas. Species diversity index was greater in restoration areas than in mining and tailings areas than in Original forests (**Figure 2**).

### 3.4 The coupling relationship between forest community structure and plant diversity

The community structure of the tree layer, shrub layer and herb layer were correlated with the diversity indices. In the tree layer, they showed significant negative correlations between the richness and the average heights, crown widths and DBH of trees (R^2^ = -0.761, *P<* 0.05; R^2^ = -0.778, *P<* 0.05; R^2^ = -0.804, *P<* 0.01). However, correlations between Shannon Wiener’s index, Simpson’s index and Alatalo’s evenness index and community structure indicators were not significant. In the shrub layer, there was also no significant correlation between species diversity indices and community structural characteristics. In the herb layer, the Alatalo evenness index had a significant negative correlation (R^2^ = -0.683, *P<* 0.05) with the mean coverage of plants. Moreover, the Pielou evenness index negatively correlated with DBH (R^2^ = -0.667, *P<* 0.05) and the mean crown width (R^2^ = -0.700, *P<* 0.05) of the trees, respectively. Mean herbaceous plant heights also highly correlated with mean tree heights (*P<* 0.01).

## 4 Discussion

### 4.1 Community structure in different operational areas

This study showed that the mining operation did not significantly affect the plant community structure in the tree layer and shrub layer. Nevertheless, the tree height, DBH and crown width were lower around the mining area, which was caused by the acidic wastewater produced by copper slag, caused a negative effect on the growth of plants (Mir et al., 2021). The maximum values of the weighted average indices of shrub height, basal diameter, and crown width were all found in the restoration area, indicating that after a period of restoration, shrubs in the restoration area have reached a level that is close to the original forest. In the herb layer, the height and coverage of the herb in the restoration area were significantly higher than those in the mining and tailings areas, with the minimum values occurring in the original forests (**Table 3**). This is due to the fact that, when comparing the different operational areas, the original forest is the most dense, resulting in a weaker light intensity for the understory plants, which affects the growth of herbaceous plants, while the restoration area grows well due to the lack of trees.

### 4.2 Species diversity in different operational areas

This study demonstrated that there were no significant difference in species diversities among different study areas (**Figure 2**), indicting that mining did not have an impact on species diversity in the region, especially in forest and shrub layer. However, species diversity seemed higher in mining and tailing area, which matched with the intermediate disturbance hypothesis that moderate anthropogenic disturbance can increase species diversity. Fang et al. (2022) conducted a study in wetland and came to a similar conclusion, they clarified the tree layer in the minimally and lightly disturbed areas had the highest Shannon Wiener and Simpson indices and relatively high community stability (Fang et al., 2022). In addition, this study also concluded that plant diversity peaked in restoration area (**Figure 2**). In original forest and mining and tailing area, increasing canopy density in the tree layer increases light limitation for herbaceous plants, resulting in the decline in herbaceous diversity (Schoonmaker and McKee, 1988; Jiménez et al., 2015), indicating that community structure should not be neglected when considering plant diversity.

### 4.3 The coupling relationship between forest community structure and plant diversity

**Figure 3:**
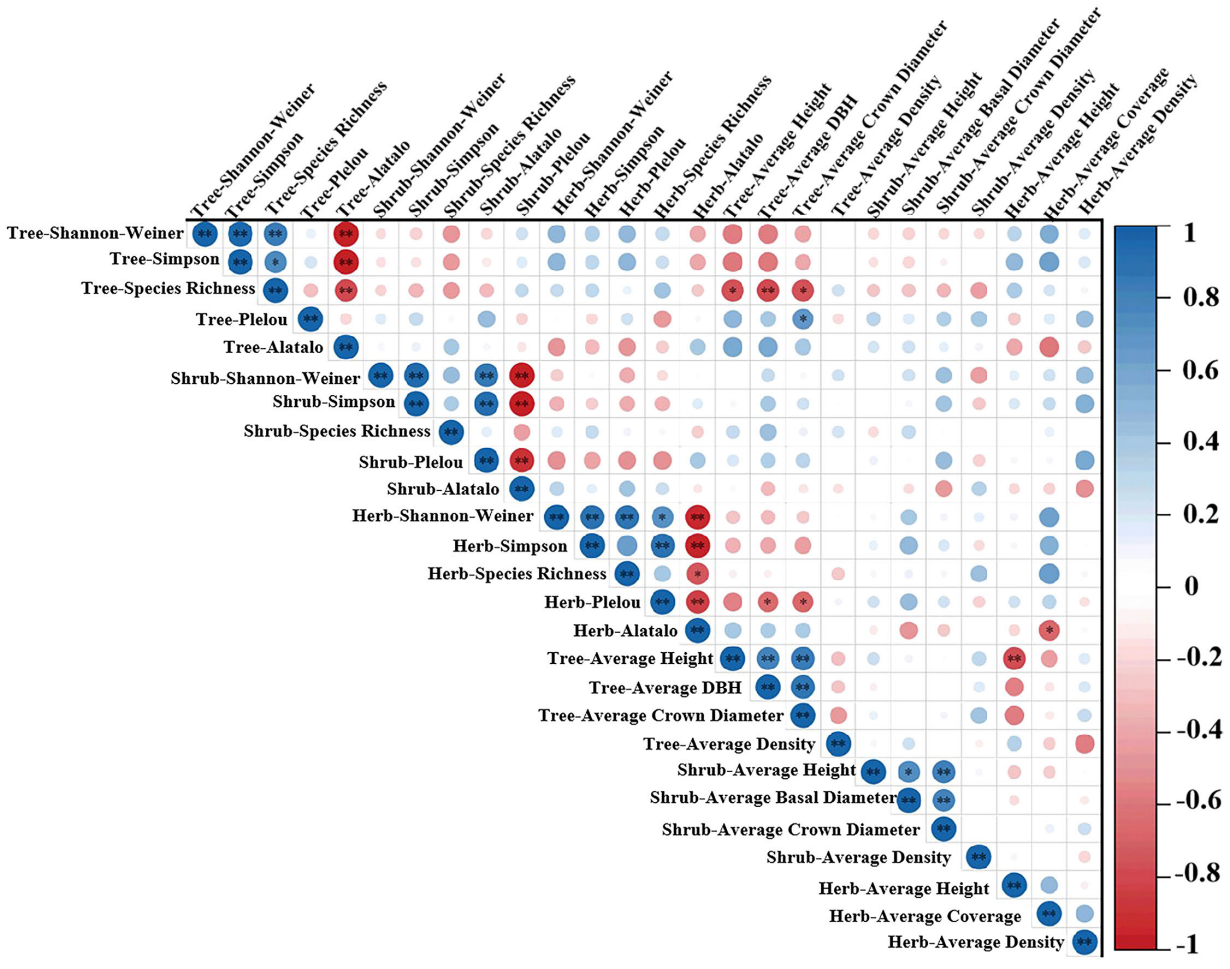
Correlation coefficient matrix of community structure and plant diversity. Significant correlation coefficients (*p<* 0.05) were highlighted in different red and blue colors. Blue represents positive correlation, while red represents negative correlation. Color intensity and size of circle are proportional to correlation coefficients (*P< 0.05; **P< 0.01).

Community structure could determine the maintenance and formation of diversity, and diversity also plays a vital role in the stability and functioning of community structure. In this study, a strong correlation between diversity and community structure was also found (**Figure 3**).

The correlations between the species richness and community structure showed that the species diversity was negatively correlated with tree growth condition, which is consistent with the results of Thompson et al. (2005). However, there are also studies that show no relationship (Kahmen et al., 2005). In addition, in the study areas, we found community structural characteristics of the shrub layer did not influence plant diversity or plant growth (**Figure 3**). In the herb layer, average cover of herb, mean DBH and crown width of trees affected herbaceous plant community diversity by influencing evenness (**Figure 3**). Similarly, Graf et al. (2019) found that forest canopy dynamics and canopy density significantly affect herbaceous plant diversity (Graf et al., 2019).

## 5 Conclusion

Through a survey of plant communities in different operating areas of gold and copper mining in the Northeast Tiger and Leopard National Park, this research elucidated the interference of mining activities on surrounding plant communities. It was found that mining did not have a significant impact on community structure and diversity in Northeast Tiger and Leopard National Park, which still keep in an intermediate level of interference. And the condition of plant community structure and diversity still need to be monitored continuously. This study provides scientific support for evaluating the impacts of open-pit gold and copper mining on forest ecosystem stability and potential threats to the habitat of Northeast Tiger and Leopard, and provides certain scientific reference value for the scientific management and policy formulation of national Northeast Tiger and Leopard parks.

## Data availability statement

The datasets presented in this article are not readily available because data are from national park. Requests to access the datasets should be directed to Xue Wang, Wangx881@nenu.edu.cn.

## Author contributions

XW: Writing – original draft. YL: Writing – review & editing. XB: Writing – review & editing, Funding acquisition. LS: Writing – review & editing, Conceptualization. HZ: Writing – review & editing, Investigation. FC: Writing – review & editing, Resources. YX: Writing – review & editing, Investigation. WL: Writing – review & editing, Methodology. YZ: Writing – review & editing, Project administration.
